# Effect of Metformin on Cardiac Metabolism and Longevity in Aged Female Mice

**DOI:** 10.3389/fcell.2020.626011

**Published:** 2021-01-26

**Authors:** Xudong Zhu, Weiyan Shen, Zhu Liu, Shihao Sheng, Wei Xiong, Ruikun He, Xuguang Zhang, Likun Ma, Zhenyu Ju

**Affiliations:** ^1^Key Laboratory of Aging and Cancer Biology of Zhejiang Province, Institute of Ageing Research, Hangzhou Normal University School of Medicine, Hangzhou, China; ^2^Key Laboratory of Regenerative Medicine of Ministry of Education, Guangzhou Regenerative Medicine and Health Guangdong Laboratory, Institute of Aging and Regenerative Medicine, Jinan University, Guangzhou, China; ^3^Department of Cardiology, the First Affiliated Hospital of USTC, Division of Life Sciences and Medicine, University of Science and Technology of China, Hefei, China; ^4^Institute on Aging and Brain Disorders, Division of Life Sciences and Medicine, University of Science and Technology of China, Hefei, China; ^5^By-Health Co. Ltd., Guangzhou, China

**Keywords:** metformin, heart aging, longevity, ECM—extracellular matrix, inflammation, mitochondrion

## Abstract

The antidiabetic drug metformin exerts pleiotropic effects on multiple organs, including the cardiovascular system. Evidence has shown that metformin improves healthspan and lifespan in male mice, yet its lifespan lengthening effect in females remains elusive. We herein demonstrated that metformin fails to extend the lifespan in female mice. Compared to 2-month-old young controls, 20-month-old female mice showed a spectrum of degenerative cardiac phenotypes alongside significant alterations in the extracellular matrix composition. Despite lowered reactive oxygen species production, long-term metformin treatment did not improve cardiac function in the aged female mice. In contrast, RNA sequencing analyses demonstrated that metformin treatment elevated the extracellular matrix-related gene while lowering oxidative phosphorylation-related gene expression in the heart. In addition, metformin treatment induced metabolic reprogramming that suppressed mitochondrial respiration but activated glycolysis (i.e., Warburg effect) in cultured primary cardiomyocytes and macrophages, thereby sustaining an inflammatory status and lowering ATP production. These findings suggest the unexpected detrimental effects of metformin on the regulation of cardiac homeostasis and longevity in female mice, reinforcing the significance of comprehensive testing prior to the translation of metformin-based novel therapies.

## Introduction

Cardiac function declines with advancing age, thereby increasing cardiovascular diseases in the geriatric population (Gude et al., 2018). Cardiac degeneration often accompanies the proinflammatory microenvironment and lowers mitochondrial function, the two most common signatures of cardiac aging (Ferrucci and Fabbri, 2018), which is attributed to a dysregulated redox system (Dai et al., 2009), altered cell composition (Spadaccio et al., 2016), aberrant mitochondrial function (Zhu et al., 2019), and (epi-)genetic abnormalities (Zhang et al., 2018). Despite lacking a consensus on the precise mechanisms that lead to degenerative cardiac phenotypes, rejuvenating the aging heart *via* targeting immune response and mitochondrial metabolism holds great promise to attenuate the functional decline.

Metformin (Met) was initially introduced for the treatment of type II diabetes mellitus in 1957, which has been then found salutary in reducing cardiovascular mortality and morbidity (Vasamsetti et al., 2015; Bergmark et al., 2019). The benefit of this old drug in cardiovascular diseases may be attributed to its classical AMPK-dependent metabolic signaling pathways (Nafisa et al., 2018) or anti-inflammatory effect (Cameron et al., 2016) *via* the suppression of NF-kB or attenuation of endothelial dysfunction *via* the inhibition of LOX-1 (Xu et al., 2013) or anti-oxidant effect *via* inhibiting TRAF3IP2 (Valente et al., 2014) and NOX4 (Sato et al., 2016). In contrast, several reports of Met-associated lactic acidosis have been documented, although the incidence is sporadic (Fitzgerald et al., 2009; Defronzo et al., 2016). A study using the myocardial infarction (MI) swine model also suggested no effect in reducing MI size by Met (Techiryan et al., 2018), reinforcing the importance of rigorously checking Met monotherapy or in combination with other medications in large animal models to facilitate the clinical translation. Nonetheless, existing study documented a life-extending effect in male mice receiving Met (Martin-Montalvo et al., 2013), suggesting that Met could be a promising candidate to help realize healthy aging. Data on whether female mice can also benefit from Met's cardioprotective and life-extending effects remain elusive.

To this end, we set to investigate the role of Met treatment in female mice. Despite a lowered reactive oxygen species (ROS) production, long-term Met treatment did not improve cardiac function in the aged female mice. Surprisingly, rather than the life-extending effect, we found a significantly shortened lifespan in aged female mice receiving long-term Met supplementation *vs*. age-matched controls. Our results reinforce the importance of carefully evaluating the Met-based strategies in a context-dependent manner to facilitate the clinical translation of novel cardioprotective therapies.

## Methods

### Animals

All procedures involving experimental animals were conducted in full accordance with and as approved by the Animal Care and Use Committee of Hangzhou Normal University. Young (2-month-old) and old (20- to 30-month-old) mice were maintained in a temperature-controlled room (22 ± 1°C) on a 12-h light/dark cycle with *ad libitum* access to food and water.

### Chemicals and Antibodies

Metformin hydrochloride was purchased from TargetMol (T0740) or Selleck (S1950). Vinculin (13901), phosphorylated AMPKα (Thr172) (2535), AMPKα antibody (5831), and anti-rabbit IgG (7074) were purchased from Cell Signaling Technology. TRIzol Reagent (15596-018) was purchased from Invitrogen. EvaGreen Supermix (172-5201AP) was purchased from Bio-Rad Laboratories Inc. PrimeScript first-strand cDNA Synthesis Kit (6110A) was purchased from TaKaRa Bio Inc. All other chemicals were purchased from Sigma-Aldrich, unless otherwise stated.

### Isolation and Culture of Primary Cells

Primary cardiomyocytes and bone marrow-derived macrophages were isolated and cultured as previously described (Trouplin et al., 2013; Zhu et al., 2019). Dulbecco's modified Eagle's medium (Hyclone) containing 4.5 g/l glucose, penicillin–streptomycin (100 IU/ml to 100 μg/ml), 10% fetal bovine serum (Gibco), and 20 ng/ml GM-CSF was used for cell culture.

### RNA Sequencing

The RNAs were isolated from heart tissues using TRI® reagent (Sigma-Aldrich) as per the manufacturers' instructions. Library construction and sequencing were performed in Novogene, China. Briefly, RNA samples for transcriptome analysis were pre-treated with DNase and processed following the Illumina manufacturer's instructions, where magnetic beads with oligo (dT) were used to isolate polyadenylated mRNA (polyA + mRNA) from the total RNA. Fragmentation buffer consisting of divalent cations was added for shearing mRNA to short fragments of 200–700 nucleotides in length. These short fragments were used as templates to synthesize the first-strand cDNA using random hexamer priming. The second-strand cDNA was synthesized using buffer including dNTPs, RNase H, and DNA polymerase I. The products were purified and resolved with QIAquick PCR Purification Kit (Qiagen) and elution buffer for end preparation and tailing A, respectively. Purified cDNA fragments were connected with sequencing adapters and gel-electrophoresed to select suitable fragments for PCR amplification. The Agilent 2100 Bioanalyzer and Applied Biosystems StepOnePlus™ Real-Time PCR System were used in the quantification and analysis of the sample library for quality control. A paired-end cDNA library was constructed and sequenced using Illumina HiSeq™ X Ten at Novogene, China. The sequencing reads were mapped to the mouse reference genome (mm9) using HISAT (Kim et al., 2015). Differentially expressed genes (DEGs) between each genotype were calculated by a standard bioinformatic analysis package, and the hierarchical clustering for DEGs between samples was generated based on DEGs. The RNAseq data have been deposited to Sequence Read Archive under the BioProject number PRJNA681488.

### Echocardiography

Cardiac function was evaluated by transthoracic echocardiography (Vevo2100, Visualsonic) in the short-axis view. M-mode images were used for measurement of fractional shortening (FS%) and ejection fraction (EF%) in lightly anesthetized mice (1–2% isoflurane mixed in 100% of oxygen, with the mice placed on a heating table to preserve the physiological body temperature). Heart rate was monitored to maintain the mice at approximately 550 bpm.

### Seahorse Cellular Flux Assay

Cells were seeded 48 h before measurement by the XFe96 Seahorse extracellular flux analyzer (Agilent). Cartridges were hydrated by a calibrant for 24 h before measurement in a non-CO_2_ incubator at 37°C. The XFe96 Seahorse instrument was utilized following the manufacturer's instructions. The results were normalized by cell number in each well and analyzed *via* Wave software (version 2.6, Seahorse Bioscience).

### Immunoblotting

Total proteins were extracted from ventricular tissues using radioimmunoprecipitation assay buffer (Applygen Technologies Inc., Beijing, China) supplemented with phosphatase and protease inhibitors (TargetMol). Equal amounts of proteins were separated by sodium dodecyl sulfate–polyacrylamide gel electrophoresis and then transferred to polyvinylidene fluoride membranes (Millipore). The membranes were reacted with a primary antibody followed by a secondary antibody. The immunoreactive bands were detected by Immun-Star WesternC chemiluminescence solutions (BioRad).

### Endurance Running Test

The mouse exercise tolerance studies were performed as previously described (Zhu et al., 2019). All mice were acclimated to the treadmill system before the running test. The exercise regimen commenced at a speed of 15 m/min, with an inclination of 7°. The mice were considered to be exhausted and removed from the treadmill following the accumulation of 10 or more shocks (0.1 mA) per minute for two consecutive minutes. The electric shock times were registered within a 15-min running period.

### Quantitative Polymerase Chain Reaction

The transcriptional expression levels of the targeted genes were assayed by quantitative polymerase chain reaction (qPCR) using the following primer sequences: Nppa-F: 5′-TTCTTCCTCGTCTTGGCCTTT-3′, Nppa-R: 5′-GACCTCATCTTCTACCGGCATCT-3′, Nppb-F: 5′-CACCGCTGGGAGGTCACT-3′, Nppb-R: 5′-GTGAGGCCTTGGTCCTTCAAGGTCACT-3′, Myh6-F: 5′-GTGACCATAAAGGAGGACCAGG-3′, Myh6-R: 5′-CCCGAGTAGGTATAGATCATCC-3′, Myh7-F: 5′-TTCATCCGAATCCATTTTGGGG-3′, Myh7-R: 5′-GCATAATCGTAGGGGTTGTTGG-3′, Col1a1-F: 5′-CGAAGGCAACAGTCGCTTCA-3′, Col1a1-R: 5′-GGTCTTGGTGGTTTTGTATTCGA-3′, Col3a1-F: 5′-GAACCTGGTTTCTTCTCACCC-3′, Col3a1-R: 5′-TCATAGGACTGACCAAGGTGG-3′, Ppargc1a1α-F: 5′-AAGTGTGGAACTCTCTGGAACTG-3′, Ppargc1a1α-R: 5′-GGGTTATCTTGGTTGGCTTTATG-3′, Tfam-F: 5′-GGAATGTGGAGCGTGCTAAA-3′, Tfam-R: 5′-GGTAGCTGTTCTGTGGAAAATCG-3′, Atp5b-F: 5′-ACGTCCAGTTCGATGAGGGAT-3′, Atp5b-R: 5′-TTTCTGGCCTCTAACCAAGCC-3′, β-actin-F: 5′-CAGCCTTCCTTCTTGGGTAT-3′, and β-actin-R: 5′-TGGCATAGAGGTCTTTACGG-3′. Total RNA was extracted using Trizol reagent (Life Technologies) according to the manufacturer's instructions. Reverse transcription and qPCR were performed using the PrimeScript first-strand cDNA Synthesis Kit (TaKaRa Bio) and EvaGreen Supermix (Bio-Rad) as per the manufacturers' instructions. The cycle threshold value determined for each RNA was normalized to the β-actin content to indicate the relative RNA level.

### Histological Analysis

The hearts were fixed with 4% paraformaldehyde and embedded in paraffin, and 6-μm-thick sections were stained with Masson's trichrome as per the manufacturer's instructions (#GP1032, Servicebio). Images were acquired using a Panoramic automated slide scanner.

### Flow Cytometry

ROS levels were analyzed with an LSRFortessa^TM^ cell analyzer (BD Biosciences) as described previously (Zhu et al., 2019).

### NAD^+^/NADH Ratio Measurement

Left ventricular tissues were freshly excised and snap-frozen for the measurements. NAD^+^/NADH ratio was analyzed with a commercial NADH/NAD quantification kit (K337-100; Biovision) as per the manufacturers' instructions.

### Statistics

All experimental results are representative of repeated experiments. Statistical analyses were performed using Prism 7 (GraphPad Software Inc.) and Image J software (version 1.48). The data are represented as mean values ± the standard error of the mean. Unpaired Student's *t*-test (two-tailed) was used to compare two normally distributed data sets. Two-way ANOVA was used for seahorse analyses. Survival analyses were performed using the Kaplan–Meier method, and the significance of differences between survival curves was calculated using the log-rank (Mantel–Cox) test. A *P* value < 0.05 was considered to be statistically significant.

All data from these experiments are available from the corresponding author upon reasonable request.

## Results

### Phenotyping the Age-Associated Cardiac Degeneration in Female Mice

**Figure 1:**
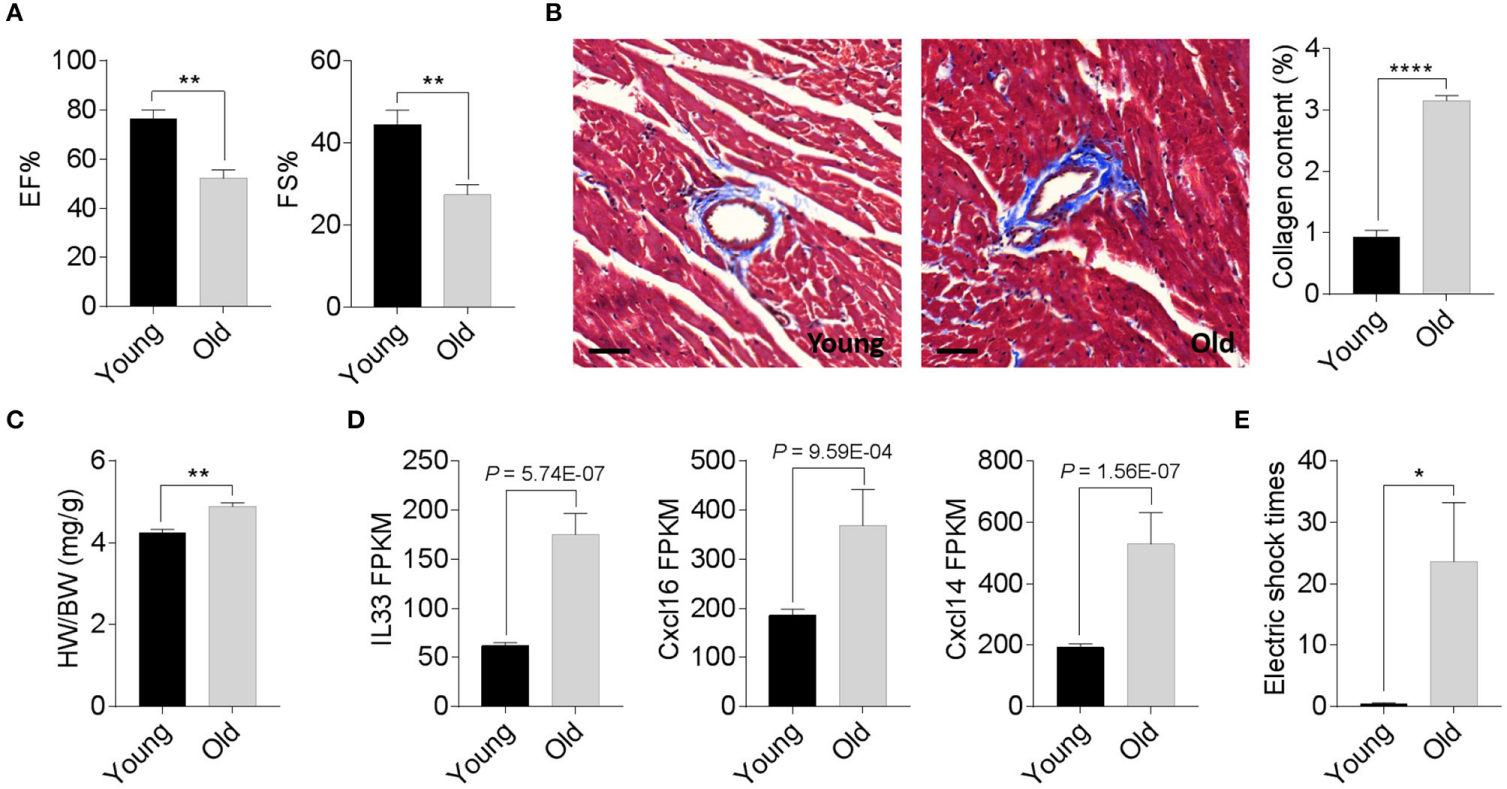
Phenotypic cardiac degeneration in old mice. **(A)** Measurements of ejection fraction (EF%) and fractional shortening (FS%) in 2-month-old and 20-month-old wild-type (WT) female mice; *n* = 5 per group, **P < 0.01. **(B)** Representative images of Masson's trichrome staining and collagen content quantification in 2-month-old and 20-month-old WT female hearts; *n* = 5 for each group, bar = 50 μm, ****P < 0.0001. **(C)** Quantification of heart weight-to-body weight ratio in 2-month-old and 20-month-old WT female mice; *n* = 5 for each group, **P < 0.01. **(D)** Relative inflammatory cytokine comparisons between 2-month-old and 20-month-old WT female hearts. Data derived from RNA sequencing analyses, *n* = 3 per genotype. The *P* values are displayed. FPKM, fragments per kilobase of exon model per million reads mapped. **(E)** Exercise tolerance test in 2-month-old and 20-month-old WT female mice. The mice were subjected to treadmill exercise once a day for four consecutive days; *n* = 5 per genotype, *P < 0.05.

We compared the degenerative cardiac signatures of 2-month old C57BL/6 (young) and 20-month-old (old) female mice with identical breeding conditions. The echocardiographic assessment confirmed a significantly reduced ejection fraction and fraction shortening in old female mice compared to those of young mice (Figure 1A). Histological staining also found more cardiac collagen deposition (Figure 1B) in the aged females, which coincided with an increased heart weight-to-body weight ratio (Figure 1C) and augmented inflammatory gene expression (Figure 1D). To evaluate whether these age-related degenerative indices link to impaired exercise capacity, we next examined the 15-min-run tolerance between young and old females. All mice were acclimated to the treadmill system with a gradual increment of running speed (from 7.5, 10, 12.5 to 15 m/min). Young female mice exhibited an excellent exercise capacity such that all were able to run for more than 30 min. In contrast, aged mice could not maintain the speed and struggled to catch up with the set speed, thus experiencing a high frequency of electric shock (Figure 1E). These data suggest that 20-month-old female mice have already exhibited phenotypes of age-associated cardiac degeneration, although the outer appearance is far from advanced aging.

### Late-Life Metformin Treatment Is Detrimental to Female Mice

**Figure 2:**
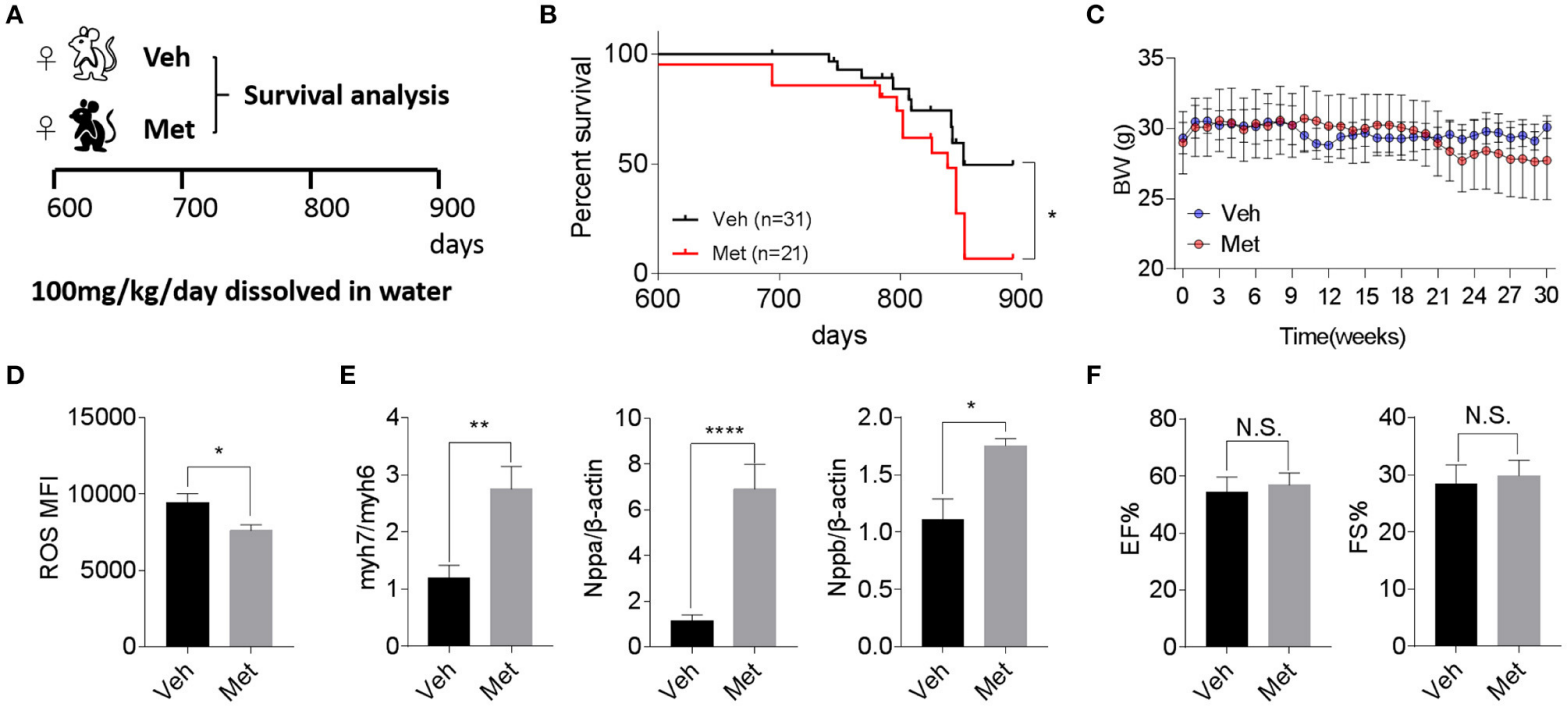
Metformin treatment is not associated with improved cardiac function and longevity in aged female mice. **(A)** The schematic image depicts the experimental procedures. Aged female wild-type (WT) mice were given water (Veh) or metformin (Met) from 600 days onwards (100 mg/kg/day oral gavage till the end of the experiment). **(B)** A shortened lifespan was seen in Met-treated female mice. Kaplan–Meier survival curves with Mantel–Cox test; *n* = 31 for Veh group and *n* = 21 for Met group, *P < 0.05. **(C)** Body mass curve comparisons between Veh and Met female mice. The mice were weighed weekly since the start of the metformin treatment. **(D)** The Met-treated mice exhibited a lower reactive oxygen species production *vs*. Veh mice; *n* = 8 for Veh group and *n* = 6 for Met group; *P < 0.05. MFI, mean fluorescent intensity. **(E)** Cardiac stress-related gene expression was upregulated in Met-treated old female mice. β-Actin was used to normalize the data; *n* = 8 for Veh group and *n* = 4 for Met group; *P < 0.05, **P < 0.01, ****P < 0.0001. **(F)** Measurements of ejection fraction and fractional shortening in old WT female mice with or without metformin treatment; *n* = 4 for Veh group and *n* = 3 for Met group. N.S., not significant.

Previous studies demonstrate that Met is cardioprotective under a pathological condition (Gundewar et al., 2009; El Messaoudi et al., 2013). Besides this, a life-extending effect of Met has been reported in male mice (Martin-Montalvo et al., 2013), prompting an assessment of whether Met could attenuate cardiac aging and likely extend the lifespan in female mice. To this end, we supplemented Met (100 mg/kg/day) or water as vehicle (Veh) to 20-month-old female mice till death (Figure 2A) and monitored their body weight and survival. Unexpectedly, a shortened median lifespan (Veh: 852 days *vs*. Met: 839 days, *P* < 0.05; Figure 2B) alongside a trend of body weight decrease (Figure 2C) at late stage was seen in female mice receiving Met treatment. This Met toxicity to female mice was not due to cardiac oxidative stress since Met treatment lowered the ROS production (Figure 2D). However, cardiac stress indices, including Myh7/Myh6, Nppa, and Nppb, were all elevated in the Met group compared to the Veh group (Figure 2E), although the ejection fraction and fraction shortening were unchanged between the two cohorts (Figure 2F).

### Metformin-Treated Mice Exhibit Altered Transcriptomics

**Figure 3:**
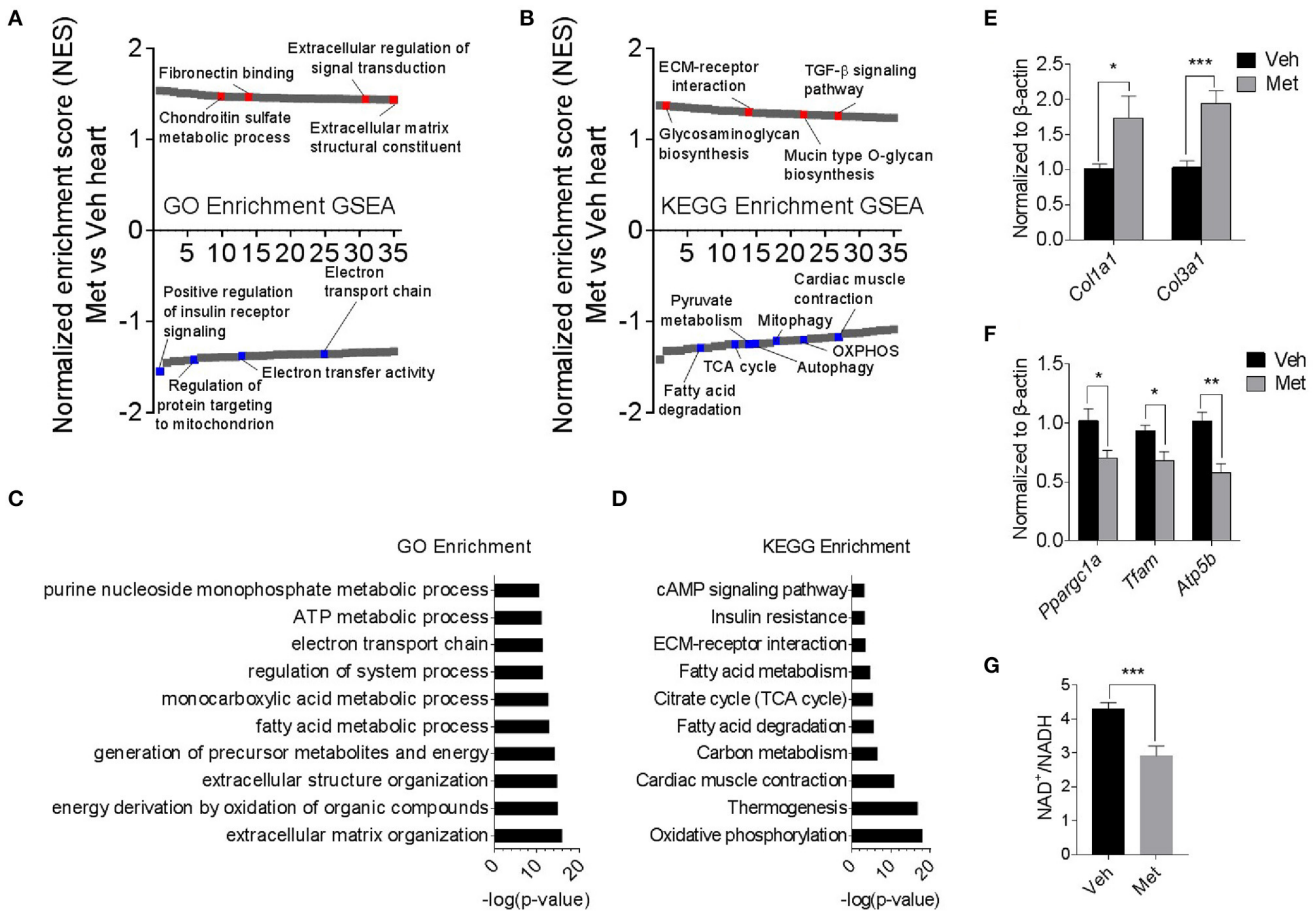
Transcriptomics analyses in old female mice with or without metformin treatment. **(A,B)** Gene set enrichment analysis demonstrated 35 TOP up- and downregulated signaling pathways in the ventricular tissue of old Met mice, compared to Veh mice. Gene Ontology enrichment and Kyoto Encyclopedia of Genes and Genomes enrichment were used for the analysis of RNA sequencing data, respectively. Signaling pathways were ranked on the basis of normalized enrichment scores (NESs); positive and negative NESs indicate down- or upregulation in aged ventricular tissue, respectively. Specific pathways related to cardiac function were highlighted in red and blue. **(C,D)** GO and KEGG enrichment-based pathway analysis of Met and Veh hearts. The results were expressed as -log (*P*-value). **(E,F)** qPCR validation of collagen synthesis-related and mitochondrial-related gene expression in Veh and Met hearts. β-Actin was used to normalize the data; *n* = 8 for Veh group and *n* = 4 for Met group. *P < 0.05, **P < 0.01, ***P < 0.001. **(G)** NAD^+^/NADH ratio was downregulated in Met-treated hearts; *n* = 4 for each group in triplicate. *P < 0.05, ***P < 0.001.

To explore the underlying mechanism of Met toxicity, we performed RNA sequencing to compare the transcriptional changes in the old heart with or without Met. Gene set enrichment analysis (GSEA) demonstrated many significantly enriched signaling pathways in the ventricular tissue of old Met mice compared to Veh mice. Gene Ontology (GO) enrichment and Kyoto Encyclopedia of Genes and Genomes (KEGG) enrichment indicated that Met treatment caused a reduction in mitochondrial respiratory genes, concurrent with an increase of extracellular matrix-related genes (Figures 3A,B). In line with this, extracellular matrix organization and oxidative phosphorylation topped in GO enrichment and KEGG enrichment, respectively (Figures 3C,D). qPCR analyses further validated the RNA sequencing findings as evidenced by augmented collagen synthesis-related genes (*Col1a1* and *Col3a1*; Figure 3E) and downregulated mitochondrial-related genes (*Ppargc1a, Tfam*, and *Atp5b*; Figure 3F). Moreover, Met-treated hearts exhibited a lowered NAD^+^/NADH ratio compared to Veh hearts (Figure 3G). These data together suggest that Met supplementation to age female mice may cause mitochondrial dysregulation and abnormal collagen biosynthesis.

### Metformin Toxicity Causing Metabolic Reprograming Is AMPK Independent

**Figure 4:**
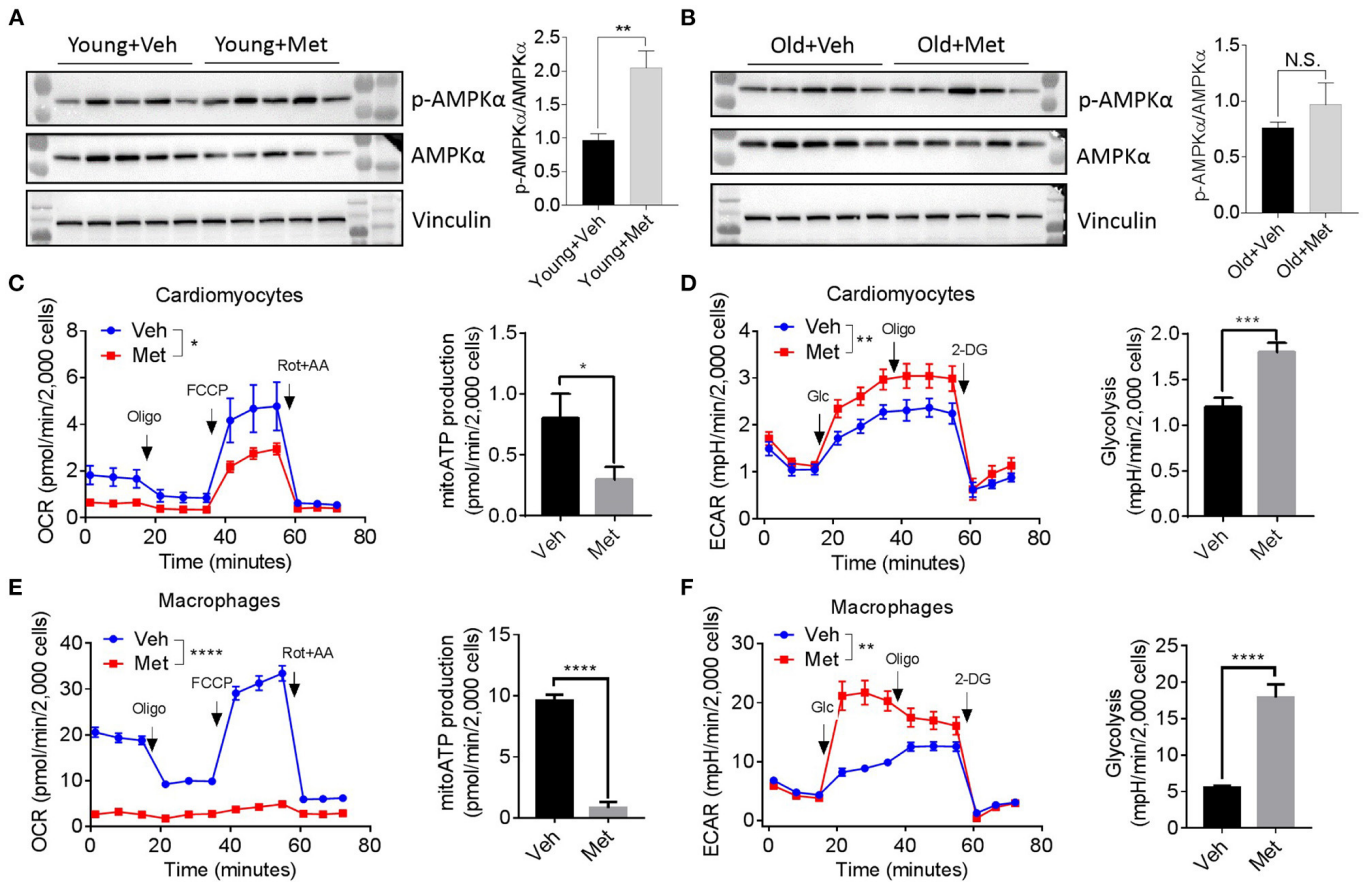
Metformin treatment fails to activate AMPK but triggers metabolic reprogramming. **(A,B)** The effect of activating AMPK *via* metformin was blunted in Met-treatment old hearts. Vinculin was used as loading control. Phosphorylated AMPK (p-AMPKα)/AMPKα ratio indicated the activation status of AMPK; *n* = 5 per group, **P < 0.01; N.S., not significant. **(C)** Mitochondrial respiration and mitochondrial ATP (mitoATP) production were measured in primary cardiomyocytes from old Veh or Met mice. Oxygen consumption rates were presented as pmol/min per 2,000 cells; *n* = 12 per group, two-way ANOVA, *P < 0.05. **(D)** Non-mitochondrial respiration and glycolytic level were measured in primary cardiomyocytes from old Veh or Met mice. Extracellular acidification rates (ECAR) were presented as mpH/min per 2,000 cells; *n* = 10 per group, two-way ANOVA; **P < 0.01, ***P < 0.001. **(E)** Mitochondrial respiration and mitochondrial ATP production were measured in primary bone marrow-derived macrophages from old Veh or Met mice. Oxygen consumption rates were presented as pmol/min per 2,000 cells; *n* = 9 per group, two-way ANOVA, ****P < 0.0001. **(F)** Non-mitochondrial respiration and glycolytic level were measured in primary bone marrow-derived macrophages from old Veh or Met mice. ECAR were presented as mpH/min per 2,000 cells; *n* = 9 per group, two-way ANOVA, **P < 0.01. Oligo, oligomycin; FCCP, carbonyl cyanide 4-(trifluoromethoxy)phenylhydrazone; Rot, rotenone; AA, antimycin A; Glc, glucose; 2-DG, 2-deoxy-D-glucose.

Since several lines of evidence demonstrate that AMPK is central to metformin's effect (Zhou et al., 2001), we assessed AMPK activation in aged hearts *via* detecting AMPKα phosphorylation at Thr172. Consistent with previous findings, the young mice receiving Met activated the phosphorylation of AMPKα at Thr172, thereby increasing the p-AMPK/AMPK ratio (Figure 4A). However, the old hearts failed to activate AMPK upon Met treatment (Figure 4B), suggesting a possible mechanism that leads to the loss of the life-extending effect in aged females. Moreover, Met incubation drastically inhibits mitochondrial respiration and ATP production, while it increased glycolysis in primary cultured primary cardiomyocytes (Figures 4C,D) and bone marrow-derived macrophages (Figures 4E,F) from aged female mice. Collectively, these data indicated that metformin induced metabolic reprogramming that suppressed mitochondrial respiration but activated glycolysis (i.e., Warburg effect), thereby sustaining a relatively low-ATP and proinflammatory microenvironment.

## Discussion

In this study, we report an unexpected Met toxicity to late-life female mice, where Met treatment fails to extend the lifespan and downregulates mitochondrial respiration. One previous study has documented that early-life Met treatment (starting from 3 months old) extended the lifespan in female SHR mice (Anisimov et al., 2008). A subsequent investigation compared the effects of different Met treatment time windows on the lifespan of female SHR mice (Anisimov et al., 2011). Interestingly, an identical dosage of Met intervention started at an early life (3 months old) increased the mean lifespan by 14%, but the salutary life-extending effect was time-dependently abolished when starting Met treatment at the age of 9 or 15 months (Anisimov et al., 2011). Despite the difference between the WT and SHR female mice, their studies and our current finding indicate that Met's effect on the lifespan is age dependent. In addition, Met was reported to exert various effects primarily in an AMPK-dependent manner, yet we did not detect any change in AMPK phosphorylation, suggesting that the loss of Met-based benefits to aged female mice is possibly AMPK independent.

Although Met was found to extend the healthspan and lifespan in mice through elevating the AMPK activity (Martin-Montalvo et al., 2013), we failed to observe such a lifespan-promoting effect in our experimental setting. This effect is reminiscent of the very recent finding that occurs with Met supplementation in *Caenorhabditis elegans*, where Met caused a lethal ATP exhaustion (Espada et al., 2020). Consistently, we also observed a decreased ATP in Met-treated cardiomyocytes, although the ejection fraction was not changed between the aged Veh and Met mice. Given that ATP decline is often associated with cardiac dysfunction, a possible explanation is that our aged female mice fed Met resembled a mouse model of heart failure with preserved ejection fraction (HFpEF), a most common form of heart failure in patients older than 65 years (Kitzman et al., 2001; Kumar et al., 2019), predominantly females (Wintrich et al., 2020). Indeed decreased ATP with preserved ejection fraction is found in patients of HFpEF. Mitochondrial deficiency of ubiquinol resulting in decreased ATP synthesis has been documented as a possible mechanism for the lowered ATP in HFpEF patients (Pierce et al., 2018). Moreover, cardiac cell types, extracellular matrix (ECM) composition, and detection methods may also affect the EF and ATP readouts. Further investigation is warranted to clarify how these factors may affect the action of Met under different circumstances.

The ECM has been long studied in the regulation of cardiac homeostasis (Bowers et al., 2010). Both structural and non-structural ECM proteins create strength and plasticity that not only provide mechanical scaffolds but also dynamically participate in physiological and pathological signaling. For instance, cardiac ECM proteins accumulate during aging, pressure overload, and myocardial infarction, thereby regulating compensatory remodeling and repair. Conversely, ECM dysregulation promotes the onset of oxidative stress and inflammation, leading to adverse cardiac remodeling and decompensation (Frangogiannis, 2017). We found an increased cardiac ECM deposition during aging, whereas collagen expression was inversely downregulated, which is in agreement with the previous literature (Meschiari et al., 2017), implicating a post-transcriptional regulation of collagen content that exists with age. We further revealed that Met treatment upregulated ECM-related transcripts, concurrent with increased proinflammatory glycolysis in the macrophages. Although we cannot exclude the possibility that abnormal ECM drives immunometabolic reprogramming, Met may disturb the ECM network in the aged heart through exacerbating mitochondrial complex I inhibition, thereby activating macrophages (Fontaine, 2018).

There are some limitations to the study. The goal of this study was to investigate whether Met exerts protective effects in myocardial metabolism and longevity in murine models, especially in females. Given the unexpected lifespan-shortening outcome in our current study and a recent worm study (Espada et al., 2020), as well as the marginal benefit of Met treatment in a MI swine model (Techiryan et al., 2018), it is obligatory to perform double-blinded, placebo-controlled clinical trials to extrapolate animal-based findings to human cardiovascular aging/diseases. Besides this, with the limited number of the aging mice cohort, we only tested one dosage at a one-time window. The methods of Met delivery, dosage, intervention time point, and age factors may lead to different consequences. In particular, acute MI causes severe fibrotic scar formation, with patients of renal impairment showing a contraindication to metformin usage due to the increased risk of lactic acidosis. Thus, the effect of Met-mediated ECM remodeling may differ from that in the chronic aging process.

In summary, our data indicated that Met did not improve the cardiac function and lifespan in old female mice. Despite the fact that the multiple salutary effects of Met pave the way to combat aging-related diseases, a systematic evaluation of Met's cardiac and life-extending effect in the late life of individuals should be considered.

## Data Availability Statement

The RNAseq data have been deposited to Sequence Read Archive (SRA) under the BioProject number PRJNA681488.

## Ethics Statement

The animal study was reviewed and approved by the Animal Care and Use Committee of Hangzhou Normal University.

## Author Contributions

XZhu and ZJ initiated the study and developed the concept of the paper. XZhu, LM, and ZJ designed the experiments. XZhu, WS, ZL, SS, RH, and XZha performed experiments. XZhu, WS, ZL, SS, WX, RH, and XZha analyzed and interpreted the data. XZhu, LM, WX, and ZJ wrote the manuscript. All authors contributed to the article and approved the submitted version.

## Conflict of Interest

The authors declare that the research was conducted in the absence of any commercial or financial relationships that could be construed as a potential conflict of interest.
